# Liquor Advertising

**Published:** 1917-03

**Authors:** 


### Liquor Advertising.

There are at present two bills pending before congress which deserve serious consideration. Both apply to all forms of advertisements, including those appearing in periodicals. The Randall bill (H. R. 18986) provides that no such advertisement, or periodical containing it, shall be mailed to anyone but a licensed manufacturer of or dealer in liquors. The Bankhead bill (S. R. 4429) provides merely that such matter shall not be mailed to any state or territory where the law at any time prohibits the advertisement or solicitation of orders for liquors.

It is an axiom of democratic government that the law shall not interfere with individual liberty although, generally and in regard to this particular issue, the principle of safeguarding society as a whole leaves it an open question whether the use of alcohol as a beverage is an individual right or whether it comes properly within the class of action detrimental to the safety of persons and property.

It is also an axiom that any law contrary to public opinion and actual usage, will be inefficient, will lead to marked injustice in its inforcement and to blackmail. The actual facts are that the year 1916, marked by great gains in anti-alcoholic voting, was also marked by a tremendous increase in the use of strong liquors over all past records and by only a slight, fluctuating decrease in the use of milder alcoholic beverages.

The proposed national legislation is, in accordance with these general principles, either right or wrong—on the face of the matter decidedly wrong and we make this statement in spite of strong personal and hereditary anti-alcoholic opinions. If we concede that the qualifications expressed allow the general tendencies of these bills to be considered as right, either of them is objectionable as striking at a branch instead of at the root of the evil. Or, to use a still more accurate figure of speech, either is objectionable as maiming an objectionable animal, and producing suffering and disease, instead of killing it mercifully at one blow in a vital spot.

So far as direct circular advertising is concerned, speaking either from the standpoint of a busy individual or with the bias of a publisher, we would welcome the interdiction not only of liquor advertising by mail, but of all forms. Such advertising is, in at least 95% of cases, a nuisance to the recipient and a handicap to the postal service in caring for useful mail. Whether it aids or injures the postoffice, from the purely financial aspect, we do not know. Circular advertising is the most expensive and the least efficient to the advertiser, except under particular conditions, as for example when the market is found among persons of leisure, who have practically no correspondence and who are thrilled with delight even at the receipt of a circular letter or card. It has, generally speaking, the sole, specious advantage of lending itself to statistics, whereas legitimate advertising in periodicals must be judged according to more general results. But, though as an individual or a publisher, we would welcome the elimination of advertising matter from the mail, we would not approve of legislation to this end, for liquors or anything else, for the reason that we believe that no one would seek to have his own interests or even his own conceptions of ethical and moral issues forced on others by law. The general action of economic law, argument as to matters of judgement and of individual conceptions of right and wrong are in the long run, far safer and saner methods of securing desired ends. At any rate, law should follow and formulate public opinion and practice, rather than precede and force it.

So far as advertisements in periodicals are concerned, the two bills are practically one. No publication worthy the name can so assort its circulation and control its secondary distribution by private individuals, or subdivide its editions to comply with the apparently more lenient provisions of the Bankhead bill, especially when we consider that both bills provide a penalty up to $1000 fine plus two years' imprisonment for the first offense.

From the limited view of medical journalism, these bills are especially objectionable. Only a minority of extremists deny that alcoholic liquors have a place of importance in therapeutics. Certain beliefs as to tonic and nutritive value have been largely discarded on account of convincing scientific demonstration. The range of alcoholic therapeutics has been narrowed as compared with the standards not only of ancient but of recent medical history. But, the great majority of the medical profession, even including men like the writer who are absolutely opposed to the non-medicinal use of alcohol, still rank alcohol and its preparations among the important and indispensable drugs. In common with all other drugs, it is practically necessary that they should be advertised to the medical profession, at least, and such a practice by constantly emphasizing the proper field of alcohol for internal use, is one of the greatest factors available in curtailing its improper use. Yet neither of these bills makes allowance for this incontrovertible fact. On the contrary, both of them practically demand that this drug and its preparations shall be excluded from their only medium of publicity which is generally admitted; they thus eliminate one of the most powerful and most easily applied methods of inforcing proper standards of quality; by implying private sources of information as the sole available one, they virtually insist that the treatment of disease, in this particular shall ultimately become surreptitious. Yet there are obvious objections to inserting in either of the bills a special exemption of medical publications. Not to mention the general objections to such limitation of effect, we would speedily have a crop of fictitious medical journals emanating from interested sources, that would almost inevitably tend to throw the whole matter of quack medicine back where it was a quarter of a century or more ago.

